# Cancer Screening Test Use — United States, 2013

**Published:** 2015-05-08

**Authors:** Susan A. Sabatino, Mary C. White, Trevor D. Thompson, Carrie N. Klabunde

**Affiliations:** 1 Division of Cancer Prevention and Control, National Center for Chronic Disease Prevention and Health Promotion, CDC; 2 Division of Cancer Control and Population Sciences, National Cancer Institute

Regular breast, cervical, and colorectal cancer (CRC) screening with timely and appropriate follow-up and treatment reduces deaths from these cancers. *Healthy People 2020* targets for cancer screening test use have been established, based on the most recent U.S. Preventive Services Task Force (USPSTF) guidelines (1). National Health Interview Survey (NHIS) data are used to monitor progress toward the targets. CDC used the 2013 NHIS, the most recent data available, to examine breast, cervical, and CRC screening use. Although some demographic subgroups attained targets, screening use overall was below the targets with no improvements from 2010 to 2013 in breast, cervical, or CRC screening use. Cervical cancer screening declined from 2010 to 2013. Increased efforts are needed to achieve targets and reduce screening disparities.

NHIS is an annual survey of a nationally representative sample of the civilian, noninstitutionalized U.S. population. The Sample Adult file was used, for which one adult was selected randomly from each family to provide information, and the Person and Imputed Income files. The 2013 sample adult response rate was 61.2%. Data from the 2013 NHIS survey (2) were used to examine recent breast, cervical, and CRC screening, defined according to USPSTF recommendations: mammography within 2 years among women aged 50–74 years, Papanicolaou (Pap) test within 3 years among women aged 21–65 years without hysterectomy, and either fecal occult blood test (FOBT) within 1 year, sigmoidoscopy within 5 years and FOBT within 3 years, or colonoscopy within 10 years among respondents aged 50–75 years, respectively.* The overall proportions of persons screened were presented as crude percentages and age standardized to the 2000 U.S. standard population. Screening use was compared by sociodemographic and access factors. Insurance includes public or private health care coverage, but excludes Indian Health Service coverage or single service plans (i.e., that pay for only one type of service). *Healthy People 2020* baseline estimates are based on 2008 NHIS data (the most recent data available in 2010 when the targets were set) (1). NHIS data from 2000, 2003, 2005, 2008, 2010, and 2013 were used to evaluate changes in screening percentages over time (2). Pearson Wald F tests were used to test for any differences across years. All statistics were weighted. Relative standard errors for all 2013 estimates were <30%.

In 2013, after adjusting for age, 72.6% of women aged 50–74 years reported recent mammography (Table 1), below the *Healthy People 2020* target of 81.1% (2008 baseline 73.7%) (1). Mammography use was lower among women aged 50–64 compared with 65–74 years, and lower among Hispanics compared with non-Hispanics. Use increased with increasing education and income. College graduates and those with income >400% of the federal poverty threshold met the target. Mammography use was lowest among those lacking insurance (38.5%) or a usual source of care (29.7%). Publicly insured women also were less likely to report screening than privately insured women. Mammography use was stable during 2000–2013 (p = 0.10) (Figure).

Overall, 80.7% of women aged 21–65 years reported a recent Pap test (age-adjusted), below the *Healthy People 2020* target of 93.0% (2008 baseline 84.5%) (1). Pap test use was lower for Asians, Hispanics, women aged 51–65 years, and foreign-born women. Uninsured and publicly insured women also were less likely than privately insured women to report screening. Use increased with increasing education and income. Use was lowest among women without a usual source of care (62.1%) or insurance (62.0%). Pap test use declined significantly by 5.5 percentage points from 2000 to 2013 (p<0.001) (Figure).

Overall, after adjusting for age, 58.2% of respondents aged 50–75 years reported recent CRC tests (Table 2), below the *Healthy People 2020* target of 70.5% (2008 baseline 52.1%) (1). CRC test use was lower among Asians and all Hispanic subgroups except Puerto Ricans compared with white and non-Hispanic respondents respectively. Use was lower among respondents aged 50–64 years (52.8%) compared with 65–75 years (69.4%) and increased with increasing education and income. Use was slightly lower among men than women (p = 0.047) and lower among foreign-born than U.S.-born respondents. Screening was particularly low among those without a usual source of care (17.8%) or insurance (23.5%). Publicly insured respondents also were less likely to report screening than privately insured respondents. Overall CRC test use increased significantly by 24.6 percentage points from 2000 to 2013 (p<0.001) (Figure). Use increased in every year assessed during 2000–2010, but not in 2013. This was true for men and women.

## Discussion

Progress toward meeting *Healthy People 2020* cancer screening targets was not observed in 2013 compared with 2010. Mammography use remained essentially stable, Pap test use declined, and CRC test use was essentially unchanged. Some subgroups attained or neared 2020 targets. The proportion of women in the highest education and income groups who were screened for breast cancer exceeded the target; the percentage of privately insured women screened was near the target value. The proportion of persons aged 65–75 years who were screened for CRC also was near the target value. Those furthest below targets were generally those without insurance or a usual source of care. For these groups, screening use was 42–53 percentage points below breast and CRC screening targets, and approximately 30 percentage points below the cervical cancer screening target. Reported screening for all three cancers was similar between whites and blacks and lower for Hispanics, with variation among racial and ethnic subgroups.

Those without insurance or usual sources of care have experienced persistent large screening disparities (3–8). Findings from the 2000 NHIS survey identified these groups as among those least likely to be up-to-date with and experiencing the greatest disparities in breast, cervical, and CRC screening (7). Based on 1987 and 1992 NHIS data, Pap test use among women aged ≥25 years was similar to these 2013 findings for those lacking a usual source of care or insurance (58% versus 62% and 65% versus 62%, respectively) (7). Moreover, although CRC test use increased from 2000 to 2008 for the uninsured aged 50–64 years and those without a usual source of care, use was low (16%–20%) and 35–40 percentage points lower than other groups (9). These 2013 data also show low screening use in these groups with disparities of similar magnitude. Only general comparisons across studies are possible because screening estimates might vary because of differences in samples, survey questions, screening definitions and recommendations over time. This trend analysis used consistent sample and screening definitions.

There are financial and nonfinancial barriers to receiving preventive services. The Affordable Care Act helps reduce financial barriers both by increasing access to insurance and by eliminating cost-sharing for breast, cervical, and CRC screening (among other preventive services) for many insured persons (10).† The National Breast and Cervical Cancer Early Detection Program§ and the Colorectal Cancer Control Program¶ reduce barriers by providing free or low-cost screening and linkages to diagnostic services for uninsured and underinsured low-income adults. The Colorectal Cancer Control Program also promotes screening through use of evidence-based interventions and health care system changes.

Efforts are needed to understand why screening percentages are not increasing, and, for Pap tests, are decreasing. In 2012, screening every 5 years with a combination of Pap and human papillomavirus (HPV) tests also was included as a screening option for some women aged 30–65 years. It is unknown whether screening intervals might have been lengthened for some women after the 2012 updated recommendation, and if so, whether this might have contributed to decreased screening use as measured in the 2013 findings. Information about HPV testing was not available. No changes in USPSTF recommendations for breast or CRC screening were made during 2010–2013. For CRC, USPSTF guidelines were updated in 2002 and 2008, and NHIS questions about endoscopy were modified in 2010. To what extent this might have contributed to changes in screening use prior to 2010 is uncertain. The National Colorectal Cancer Roundtable set a goal of 80% screened by 2018.** More than a 20 percentage-point improvement is needed to meet this goal. Colonoscopy is more commonly used than other recommended CRC screening options (6). Promotion of all recommended CRC testing options, including less invasive methods like home FOBT might increase use, particularly because the test completed (presumably reflecting patient preferences) varies among subgroups (6).

For this report, screening histories were examined only for persons in age groups recommended for routine screening. However, nearly one fourth of persons aged 51–65 years and 30% of those aged 65–75 years reported no recent cervical cancer and CRC screening, respectively, thus some might reach upper age limits for routine screening without adequate prior screening. Although USPSTF does not recommend routine screening for cervical cancer among average-risk women aged >65 years or for CRC among adults aged 76–85 years,†† screening might be indicated for some adults in these older groups who were not screened adequately when they were in a younger age group for which routine screening was recommended.

The findings in this report are subject to at least seven limitations. First, NHIS data are self-reported and not verified by medical records. Second, the response rate was 61%, and nonresponse bias is possible despite adjustments for nonresponse. Third, although age-adjusted percentages for screening are presented that are consistent with *Healthy People 2020* targets overall, percentages for subgroups are not age-adjusted. Fourth, Pap test data for 2003 were excluded because hysterectomy status was unknown. Fifth, screening guidelines and NHIS screening questions have changed over time. Sixth, confidence intervals were wide for some subgroups, indicating estimate imprecision. Finally, diagnostic tests rather than screening tests might have been reported by some respondents, possibly leading to overestimates of screening.

Increased efforts are needed to reach *Healthy People 2020* cancer screening targets and reduce disparities. More intensive or focused efforts might be required to overcome persistent barriers among specific population subgroups. Making available all recommended CRC screening options might increase alignment of tests with individual needs and preferences, and facilitate screening completion. Evidence-based interventions can increase screening use. Information about recommended interventions is available for communities and health systems from The Community Guide.§§ Cancer Control PLANET¶¶ provides resources for designing and implementing evidence-based programs. Such resources can help communities identify and implement effective interventions appropriate for their needs to increase use of these important services.

What is already known on this topic?

Screening is effective for detecting breast, cervical, and colorectal cancers early when the cancers can be more easily treated and deaths averted. *Healthy People 2020* established targets for breast, cervical, and colorectal cancer screening in the United States. Disparities in screening use related to several demographic and health care access factors have been observed.

What is added by this report?

The most recent data on screening use (from 2013) show no progress toward meeting *Healthy People 2020* targets for cancer screening. Mammography use in women aged 50–74 years was 72.6% (target 81.1%), Pap test use in women aged 21–65 years was 80.7% (target 93.0%), and CRC screening in persons aged 50–75 years was 58.2% (target 70.5%). Compared with 2000, mammography use was unchanged, Pap test use was lower and CRC screening was higher, although unchanged since 2010. Persons without a usual source of care or insurance generally were furthest below *Healthy People 2020* targets.

What are the implications for public health practice?

Progress toward *Healthy People 2020* targets requires efforts to increase breast, cervical and colorectal cancer screening use overall. Evidence-based interventions, such as client and provider reminders and others, can increase screening use.

## Figures and Tables

**FIGURE:**
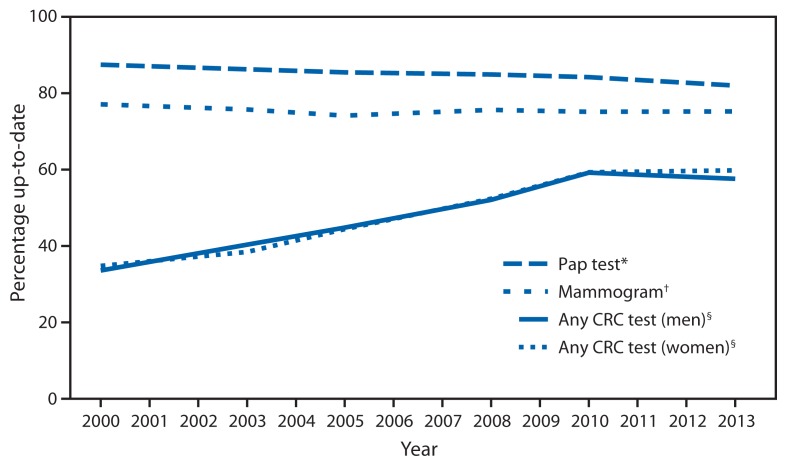
Percentage of adults up-to-date with screening for breast, cervical, and colorectal cancers by test, sex, and year — United States 2000–2013 **Abbreviations:** CRC = colorectal cancer; Pap = Papanicolaou. **Source:** National Health Interview Survey, 2000, 2003, 2005, 2008, 2010, and 2013. ^*^ Among women aged 21–65 years with no previous hysterectomy. Pap test data for 2003 were excluded because hysterectomy status was not ascertained in that year. ^†^ Among women aged 50–74 years. ^§^ Among persons aged 50–75 years.

**TABLE 1:** Percentage of women who received recent breast and cervical cancer screenings, by selected demographic and access to care characteristics — National Health Interview Survey, United States 2013

Characteristic	Breast cancer			Cervical cancer		
	
	Mammogram ≤2 years			Pap test ≤3 years		
	
	No.	%*	(95% CI)	No.	%*	(95% CI)
**Overall**						
Crude	7,012	72.5	(71.2–73.9)	11,857	80.5	(79.6–81.5)
Age-adjusted†	7,012	72.6	(71.2–73.9)	11,857	80.7	(79.7–81.6)
**Race** §		p = 0.996			p<0.001	
White	5,386	72.6	(71.0–74.1)	8,683	81.2	(80.1–82.2)
Black	1,179	72.6	(68.8–76.1)	2,082	82.2	(80.0–84.3)
American Indian/Alaska Native	84	73.4	(60.0–83.5)	145	83.1	(73.8–89.6)
Asian	336	72.0	(66.4–77.0)	851	70.1	(65.8–74.0)
Chinese	66	74.4	(60.4–84.7)	188	64.0	(55.4–71.8)
Filipino	106	67.7	(56.7–77.0)	224	82.9	(76.2–88.0)
Other Asian	164	73.2	(64.2–80.7)	439	66.8	(60.6–72.5)
**Ethnicity** ¶		p = 0.001			p<0.001	
Non–Hispanic	6,135	73.2	(71.7–74.6)	9,420	81.3	(80.2–82.3)
Hispanic	877	66.5	(62.6–70.2)	2437	76.9	(74.7–78.9)
Puerto Rican	112	69.5	(60.2–77.5)	230	82.3	(76.3–87.0)
Mexican	246	63.3	(55.9–70.0)	955	73.9	(70.2–77.3)
Mexican-American	215	71.7	(63.4–78.8)	543	81.1	(76.9–84.6)
Central/South American	141	67.6	(56.9–76.7)	405	76.1	(70.5–80.9)
Other Hispanic	163	60.8	(50.9–69.9)	304	76.7	(70.7–81.8)
**Age group (yrs)**		p = 0.005			p<0.001	
21–30				3,075	79.9	(77.8–81.8)
31–40				3,118	83.1	(81.3–84.8)
41–50				2,410	82.2	(80.5–83.8)
51–65				3,254	77.6	(75.7–79.4)
50–64				4,619	71.4	(69.7–73.1)
65–74				2,393	75.3	(73.1–77.3)
**Period of U.S. residence**		p<0.001			p<0.001	
U.S.–born	5,875	73.0	(71.4–74.5)	9,247	82.2	(81.2–83.2)
In United States <10yrs	68	40.8	(25.5–58.2)	631	66.0	(61.5–70.1)
In United States ≥10yrs	1,054	71.9	(68.7–74.9)	1,943	76.7	(74.0–79.2)
**Education**		p<0.001			p<0.001	
Less than high school	1,010	59.8	(55.5–63.9)	1,532	69.8	(66.6–72.7)
High school graduate	1,936	69.1	(66.5–71.6)	2,553	75.1	(72.9–77.2)
Some college/Associate degree	2,169	72.8	(70.4–75.1)	3,787	81.4	(79.7–83.1)
College graduate	1,868	81.2	(78.7–83.6)	3,942	86.6	(85.0–88.0)
**% of federal poverty threshold**		p<0.001			p<0.001	
<139%	1,617	56.3	(53.2–59.5)	3,487	69.7	(67.7–71.5)
139%–250%	1,347	64.0	(60.4–67.4)	2,328	76.8	(74.4–79.1)
251%–400%	1,471	73.9	(70.8–76.7)	2,348	83.0	(80.8–85.0)
>400%	2,577	81.8	(79.9–83.6)	3,694	87.7	(86.4–88.9)
**Usual source of care**		p<0.001			p<0.001	
None or hospital emergency department	535	29.7	(25.1–34.7)	1,931	62.1	(59.4–64.7)
Has usual source	6,477	75.7	(74.4–77.0)	9,924	83.9	(82.9–84.8)
**Health care coverage**		p<0.001			p<0.001	
Private/Military	4,339	79.9	(78.5–81.3)	7,333	86.3	(85.2–87.2)
Public only	1,915	66.4	(63.8–68.9)	2,048	78.8	(76.3–81.1)
Uninsured	742	38.5	(34.2–43.0)	2,434	62.0	(59.5–64.5)

**Abbreviations:** CI = confidence interval; Pap = Papanicolaou.

* Weighted percentages. Overall percentages presented as crude and age–adjusted estimates. Other percentages are crude estimates.

† Age–standardized to the 2000 U.S. standard population.

§ p-value testing for differences across four primary race groups.

¶ p-value testing for differences between Hispanic and non-Hispanics.

**TABLE 2:** Percentage of men and women who received recent colorectal cancer screenings, by selected demographic and access to care characteristics — National Health Interview Survey, United States 2013

Characteristic	Colorectal cancer*		

	No.	%†	(95% CI)
**Overall**			
Crude	13,045	57.8	(56.6–59.0)
Age–adjusted§	13,045	58.2	(57.0–59.3)
**Sex**		p = 0.047	
Men	5,873	56.7	(55.0–58.3)
Women	7,172	58.9	(57.3–60.5)
**Race** ¶		p = 0.010	
White	10,135	58.4	(57.0–59.7)
Black	2,096	57.9	(54.7–61.0)
American Indian/Alaska Native	149	48.3	(36.4–60.5)
Asian	612	49.5	(44.1–54.9)
Chinese	117	52.2	(42.2–62.1)
Filipino	175	52.2	(43.3–61.0)
Other Asian	320	46.7	(39.3–54.3)
**Ethnicity** **		p<0.001	
Non–Hispanic	11,495	59.6	(58.4–60.8)
Hispanic	1,550	41.5	(38.3–44.8)
Puerto Rican	194	59.4	(50.5–67.8)
Mexican	490	32.4	(27.3–38.1)
Mexican American	342	49.0	(41.9–56.1)
Central/South American	259	36.9	(30.5–43.8)
Other Hispanic	265	41.2	(33.3–49.5)
**Age group (yrs)**		p<0.001	
50–64	8,527	52.8	(51.2–54.3)
65–75	4,518	69.4	(67.8–71.0)
**Period of U.S. residence**		p<0.001	
U.S.–born	10,996	59.9	(58.7–61.2)
In United States <10yrs	136	19.3	(12.3–28.9)
In United States ≥10yrs	1,887	48.3	(45.2–51.4)
**Education**		p<0.001	
Less than high school	2,008	43.6	(40.6–46.6)
High school graduate	3,573	53.4	(51.3–55.5)
Some college/associate degree	3,823	59.2	(57.1–61.3)
College graduate	3,596	66.7	(64.7–68.6)
**% of poverty threshold**		p<0.001	
<139%	2,891	44.2	(41.6–46.8)
139%–250%	2,445	52.6	(49.6–55.5)
251%–400%	2,736	56.0	(53.3–58.6)
>400%	4,973	65.6	(63.8–67.4)
**Usual source of care**		p<0.001	
None or hospital emergency department	1,226	17.8	(15.2–20.8)
Has usual source	11,819	61.5	(60.2–62.7)
**Health care coverage**		p<0.001	
Private/Military	8,141	63.0	(61.6–64.4)
Public only	3,438	58.7	(56.4–60.9)
Uninsured	1,435	23.5	(20.6–26.6)

**Abbreviation:** CI = confidence interval.

* Includes fecal occult blood test ≤1 year, flexible sigmoidoscopy ≤5 years and FOBT ≤3 years, or colonoscopy ≤10 years.

† Weighted percentages. Overall percentages presented as crude and age–adjusted estimates. Other percentages are crude estimates.

§ Age-standardized to the 2000 U.S. standard population.

¶ p-value testing for differences across four primary race groups.

** p-value testing for differences between Hispanic and non-Hispanics.

## Footnotes

* Available at http://www.uspreventiveservicestaskforce.org.

† Additional information available at http://www.hhs.gov/healthcare/facts/timeline/timeline-text.html.

§ Additional information available at http://www.cdc.gov/cancer/nbccedp/.

¶ Additional information available at http://www.cdc.gov/cancer/crccp/.

** Additional information available at http://nccrt.org/tools/80-percent-by-2018/.

†† Additional information available at http://www.uspreventiveservicestaskforce.org.

¶¶ Additional information available at http://cancercontrolplanet.cancer.gov/.
